# Characterization of the Interlaminar Fracture Toughness of an Additive Manufacturing Continuous Glass Fiber-Reinforced Thermoplastic Composite

**DOI:** 10.3390/polym18121438

**Published:** 2026-06-09

**Authors:** Jonnathan D. Santos, Fernando Crespo Beltrán, Mateo Berrezueta, Alexander Torres, Alex Gavilanes Álvarez, Alfredo Valarezo

**Affiliations:** 1 Análisis y Tecnología de Estructuras en Ingeniería (ATEI), Universidad Politécnica Salesiana, Cuenca 010105, Ecuador; mberrezuetaa@est.ups.edu.ec (M.B.);; 2 Department of Engineering, University of Campania “Luigi Vanvitelli”, 81031 Aversa, Italy; fernandomateo.crespobeltran@unicampania.it; 3 Institute for Energy and Materials, Mechanical Engineering Department, Universidad San Francisco de Quito, Quito 170901, Ecuador; avalarezo@usfq.edu.ec

**Keywords:** interlaminar fracture toughness, 3D-printed composite, fused filament fabrication, mechanical testing, glass fiber-reinforced polyamide

## Abstract

There is a lack of knowledge concerning the interlaminar fracture toughness of 3D-printed composite materials using both commercial filament composites and fused deposition modeling (FDM) technology from Markforged^®^. In this investigation, additive manufacturing (AM) continuous fiber-reinforced thermoplastic (cFRT) specimens have been tested to characterize the initiation and propagation of interlaminar fracture toughness in mode I (*G*_I_). Unidirectional glass fiber (GF)-reinforced polyamide 6 (PA) laminates were characterized by means of the double cantilever beam (DCB) test. These specimens were manufactured using a MarkTwo^®^ printer and tested without doublers, following a laminate configuration selected according to appropriate experimental findings reported in the state of the art, ensuring reliable fracture characterization. The experimental results exhibited repeatability and strong agreement between the modified compliance calibration (MCC) and modified beam theory (MBT) reduction methods. The resistance curve (R-curve) indicated a progressive increase in fracture resistance during crack propagation. To analyze the experienced failure mechanism during testing, the fracture surfaces of representative post-mortem DCB specimens were observed using a scanning electron microscope (SEM), revealing characteristic morphological features at two magnification levels. Moreover, representative cross-sections of the tested DCB specimens were electronically observed to analyze the interlaminar morphologies, showing an irregular and random distribution of the matrix, fiber, and voids between consecutive plies and adjacent deposited rasters. Compared with previously reported Markforged^®^ continuous fiber-reinforced systems, the GF/PA composite material exhibited intermediate initiation fracture toughness but lower propagation toughness. This study contributes to filling the existing gap in fracture toughness data for glass fiber-reinforced additively manufactured composites.

## 1. Introduction

Continuous fiber-reinforced polymer (FRP) composites have been widely employed in different sectors that play an important role in the worldwide economy, including automotives, sports, construction, defense, aeronautics, and aerospace. Their appeal lies in the combination of outstanding mechanical properties, such as a high strength-to-weight ratio, elevated stiffness, high thermal conductivity, and excellent resistance to corrosion. Traditionally, these composite materials are fabricated using conventional manufacturing techniques such as resin transfer molding (RTM), spray lay-up, vacuum bagging, filament winding, and pultrusion. However, these methods involve time-consuming procedures that require highly qualified operators, along with the use of costly equipment, tooling, and molds. Such requirements render them economically unsuitable for small-batch production or rapid prototyping. These drawbacks have prompted the development of additive manufacturing (AM) technologies, which have emerged as a reliable and cost-efficient alternative for manufacturing complex composite structures while minimizing the weight and improving the design flexibility [1,2,3].

Fused deposition modeling (FDM) is one of the most widely used AM technologies, owing to its attractive manufacturing capabilities. Its advantages include high dimensional accuracy, out-of-autoclave processing, low material waste, and easy-to-fabricate multi-material-based structures. In addition, FDM involves a wide spectrum of printers, from industrial high volumes and printing speeds to personal domestic on-desktop units [4,5]. This technology can produce short fiber-reinforced thermoplastic (sFRT) composites, which have been extensively investigated during the last decade [6,7,8,9,10]. Short fibers are incorporated into a pure raw polymeric matrix, most commonly nylon, polylactic acid (PLA), and acrylonitrile butadiene styrene (ABS) [2,11]. These discontinuous fibrous systems show limited mechanical properties for structural applications [2,3,5,12]. In contrast, continuous fiber-reinforced thermoplastic (cFRT) composites exhibit superior damage resistance and enhanced mechanical properties, including a higher tensile modulus and tensile strength and excellent interlaminar facture toughness [5,13,14,15,16].

In the literature, numerous experimental investigations have been conducted to evaluate the mechanical performance of cFRT composites under various loading conditions. Among these, the in-plane tensile test has been extensively investigated [13,17,18,19,20,21,22], while bending has attracted less research attention [15,23,24,25,26]. However, a limited number of studies have addressed the long-term response, including fatigue damage evolution [27,28,29] and creep properties [30,31], impact resistance [32], environmental effects on mechanical properties [33], and fracture toughness [34]. The fracture response at the interfaces between plies is of significant concern regarding the structural integrity of AM parts, since cFRT composites are fabricated through a robotic process involving side-by-side raster deposition and layer-by-layer ply stacking. This is a fundamental issue in AM parts, arising from the weaker bonding between consecutive deposited layers in relation to adjacent rasters along the printing plane [35]. Delamination is the most common failure mechanism in continuous-fiber composites and can occur during the service life of the part [36], which may trigger catastrophic failure [37,38].

One of the first investigations regarding the characterization of interlaminar fracture toughness in mode I, *G*_I_, for neat ABS using FDM was conducted by Aliheidari et al. [9]. The authors designed a modified compact tension (CT) specimen resembling the geometry of a double cantilever beam (DCB). The results indicated that increasing the extrusion temperature enhanced the fracture toughness, with specimens printed at 240 °C achieving values comparable to those of the bulk ABS material. Similar studies have investigated various sFRT systems under a mode I toughness response [7,8,39].

Regarding cFRT composites composed of carbon fiber (CF)-reinforced polyamide 6 (PA) from Markforged^®^, Iragi et al. [40] conducted one of the first studies to characterize the mode I and mode II interlaminar fracture toughness (*G*_II_) of CF/PA composites, conducting the DCB and end-notched flexure (ENF) tests for modes I and II, respectively. Contrary to the common tendency of FRP composites, the initiation fracture toughness in mode II, G_IIc,ini_ = 1.59 kJ/m^2^, was lower than the propagation fracture toughness in mode I, G_Ic,prop_ = 2 kJ/m^2^. The authors attributed this behavior to extensive fiber bridging during opening in mode I and limited matrix shearing during sliding in mode II tests.

Santos et al. [41] characterized *G*_I_ and *G*_II_ for the same CF/PA composite material. The results followed a similar tendency, with both the initiation and propagation fracture toughness in mode I, G_Ic,ini_ = 1.5 and G_Ic,prop_ = 1.7 kJ/m^2^, being lower than those obtained in mode II, G_IIc,ini_ = 2 and G_IIc,prop_ = 2.3 kJ/m^2^. The authors concluded that the use of doublers is unsuitable for this material type. In a related study, Polyzos et al. [42] also characterized *G*_I_ and *G*_II_ for the same CF/PA material. The two toughness values, *G*_Ic,ini_ = 1.4 and *G*_IIc,ini_ = 2 kJ/m^2^, are in good agreement with those reported by Santos et al. [41].

Goh et al. [43] were the first to explore the effects of printing parameters on the interlaminar fracture toughness performance of CF/PA composites using an open-source Hello BeePrusa on-desktop 3D printer. The highest mean fracture toughness, *G*_Ic_ = 0.9 kJ/m^2^, was obtained by combining the highest nozzle (265 °C) and printing bed (70 °C) temperatures with the lowest printing speed (7 mm/s).

He et al. [44] compared the *G*_I_ values of CF/PA composite materials between regular fabricated 3D-printed and compression molding (CM) post-processed specimens. The *G*_Ic,ini_ value of the CM specimens (0.2 kJ/m^2^) was almost two times higher than that of the regular 3D-printed ones (0.1 kJ/m^2^). In contrast, the *G*_Ic,prop_ value of the non-treated specimens (1.5 kJ/m^2^) was more than three times higher than that of the CM specimens (0.5 kJ/m^2^). This behavior was attributed to both the large amount of fiber bridging during opening and the greater presence of interfacial flaws in the non-treated specimens compared to the CM ones.

In line with the study by He et al. [44], Forbes et al. [34] characterized the *G*_I_ and *G*_II_ values of the same CF/PA composites, comparing the fracture responses between a regular 3D-printed specimen and a post-processed one. The latter was exposed to 150 °C for 30 min within a manually clamped compression steel tool. The post-processed specimen experienced higher fracture toughness in both modes I and II than the regular specimen, achieving improvements of up to 18% and 17%, respectively. These results were attributed to a reduction in the void content in the post-processed specimen (5%) with a more regular fracture surface, a certain influence on fiber alignment, and enhanced PA matrix properties from thermal exposure.

Kong et al. [45] were the first to characterize the interlaminar fracture toughness of CF/PA composites under mixed-mode loading using the single-lap shear (SLS) test; moreover, they explored the pure *G*_I_ and *G*_II_. The reported results, *G*_Ic,prop_ = 1.1 and *G*_IIc,prop_ = 0.5 kJ/m^2^, demonstrated a reduction in toughness behavior compared to the previous studies. This might be explained by the alternated stacking sequence, consisting of pairs of one or two Onyx™ (PA reinforced with 12% wt. short carbon fiber [46]) and CF plies along the specimen thickness for pure loadings and the SLS test, respectively. Additionally, the SLS test explored different interface materials and fiber orientations, identifying the cross-ply CF/CF as the best balance between strength and flexibility.

Katalagarianakis et al. [47] were the first to determine the interlaminar fracture toughness of CF/PA composite materials under a 50% mixed-mode loading ratio using the well-established mixed-mode bending (MMB) test. Similar to the study by Kong et al. [45], the authors also studied the pure *G*_I_ and *G*_II_. The fracture toughness values were *G*_Ic,ini_ = 1.4, *G*_Ic,prop_ = 1.9, *G*_IIc,ini_ = 2.1, and *G*_IIc,prop_ = 2.3 kJ/m^2^. The reported fracture toughness in the mixed-mode evaluations was 1 and 1.3 kJ/m^2^ for initiation and propagation, respectively. These two toughness values did not fall within the magnitudes of those of the pure modes I and II. The authors did not provide an explanation for this unexpected finding.

In a related investigation, Santos et al. [48] explored 25%, 50%, and 75% mixed-mode ratios of CF/PA composite materials using the MMB test. In contrast to the findings of Katalagarianakis et al. [47], the initiation (1.7 kJ/m^2^) and propagation (2 kJ/m^2^) fracture toughness values at the 50% mixed-mode ratio reported by Santos et al. [48] fell within the ranges of the pure *G*_I_ and *G*_II_ values previously discussed in [41]. However, the 25% and 75% mix ratios did not show a similar trend. The authors conducted optical and microscopic analyses to identify potential errors during the experimental campaign; however, a concluding finding was not reported.

Regarding non-carbon fiber systems, García-Guzman et al. [49] conducted both experimental and analytical investigations on the fracture responses of adhesively bonded DCB composite specimens composed of continuous glass fiber (GF)-reinforced PA, comparing trapezoidal and flat interface patterns. The initiation and propagation interlaminar fracture toughness values were higher for the trapezoidal interface than for the conventional flat one, due to the additional contribution of mode II along the trapezoidal path. Khosravani et al. [50] investigated the influence of the fiber volume fraction (Vf) on the stiffness and interlaminar strength of the same GF/PA composites using the semi-circular bending (SCB) test. The results indicated that increasing Vf triggered higher stiffness and fracture toughness capacity.

Following the exploration of different Markforged^®^ fiber systems, Dang et al. [51] investigated the influence of interface ply material configurations on the interlaminar fracture toughness using DCB test. Three combinations were compared: CF/CF, Kevlar fiber/Kevlar fiber (KF/KF), and a hybrid CF/KF interface. The non-hybrid CF/CF system led to *G*_Ic,ini_ = 0.2 kJ/m^2^ and *G*_Ic,prop_ = 0.9 kJ/m^2^, while the KF/KF interface resulted in *G*_Ic,ini_ = 0.1 kJ/m^2^ and significantly higher propagation toughness of *G*_Ic,prop_ = 3.1 kJ/m^2^. The hybrid CF/KF configuration improved the propagation and reduced the initiation toughness values (*G*_Ic,prop_ = 2.7 and *G*_Ic,ini_ = 0.1 kJ/m^2^).

Delbariani-Nejad et al. [52] studied the effects of hybridization on the interlaminar fracture toughness in mode II for the CF/CF, KF/KF, and CF/KF configurations. The authors employed the co-extrusion COMBOT-200 3D printer, depositing pre-impregnated fibers and a PA matrix at the same time. The reported initiation fracture toughness values for the CF/CF and KF/KF configurations were 2.7 and 1 kJ/m^2^, respectively, whereas the hybrid specimen exhibited 1.9 kJ/m^2^ under stable crack propagation. The propagation fracture toughness for CF/KF (19.2 kJ/m^2^) was approximately seven times higher than the lowest one (2.4 kJ/m^2^ for CF/CF). This toughening effect was primarily driven by KF, which promoted the development of a large failure process zone (FPZ) behind the crack tip from extensive fiber bridging.

Moreno-Nuñez et al. [53] studied the *G*_I_, *G*_II_, and MMB values at 50% in KF/PA composites. The specimen stacking sequence consisted of unidirectional and 90° fibers in the core, with external Onyx™ plies. The mode I propagation fracture toughness for the 90° fiber orientation (1.62 kJ/m^2^) was higher than that of the 0° one (1.06 kJ/m^2^). Similarly, the initiation MMB toughness behavior was reported as 1.3 and 1 kJ/m^2^ for the 90° and 0° reinforcement orientations, respectively. In contrast to this trend, the authors reported *G*_IIc,ini_ = 3.7 and *G*_IIc,ini_ = 5.4 kJ/m^2^ for the 90° and 0° fiber orientations, respectively.

Shih et al. [54] characterized the *G*_I_ values of CF/PA and a high-strength/high-temperature glass fiber (HSHTGF)-reinforced PA composite material, exploring three different stacking sequences with approximate fiber volume fractions (FVFs) of 19%, 23%, and 28%. The statistical analysis concluded that the fiber content, along with the stacking sequence, had no significant effect on the fracture toughness response. The reported toughness values were *G*_Ic,ini_ = 1 and *G*_Ic,prop_ = 1.8 kJ/m^2^ for CF/PA and *G*_Ic,ini_ = 1.1 and *G*_Ic,prop_ = 1.5 kJ/m^2^ for HSHTGF/PA. The experimental campaign exhibited fiber bridging and symmetric opening in both fibrous systems.

On the one hand, the mode I and mode II interlaminar fracture toughness of CF/PA composites have been investigated in several studies from different outlooks, using different procedures, and obtaining relevant findings. On the other hand, investigations focusing on mixed-mode loading characterization remain limited. However, we note a lack of fracture investigations dealing with KF/PA composites and even fewer addressing GF/PA ones. To the best of the authors’ knowledge, only two investigations have considered the fracture toughness performance of structured interface configurations in GF/PA [49,55], and the work by Shih et al. [54] determined the *G*_I_ of HSHTGF/PA. It is highly relevant to expand the current knowledge about the *G*_I_ of GF/PA, which is a key material property for improving the design feasibility of the final AM parts, particularly in ensuring structural stability against potential catastrophic delamination failure.

Accordingly, in this work, we experimentally characterized the mode I interlaminar fracture toughness of cFRT GF/PA AM composites using the DCB test. The obtained initiation fracture toughness fell within the range of previously reported toughness values for cFRT composite materials, while the propagation one exhibited lower resistance than the compared systems. Fracture surface observation of representative tested specimens was conducted using a scanning electron microscope (SEM), and the resulting fractographic images revealed specific morphologic failure mechanisms. The organization of the article is as follows. In Section 2, the materials, manufacturing process, specimen preparation, and testing protocol are described. The experimental results and discussion are presented in Section 3 and Section 4, respectively.

## 2. Materials and Methods

This section describes the manufacturing procedure and preparation process of the DCB specimens, as well as the experimental test campaign to obtain the *G*_I_ values of the unidirectional (UD) laminates.

### 2.1. Specimen Material and Manufacturing

In this study, a UD thermoplastic composite was manufactured via AM using the on-desktop MarkTwo^®^ 3D printer from Markforged^®^ (Watertown, MA, USA), consisting of the continuous glass fiber (GF)-reinforced polyamide 6 (PA), referred to as GF/PA. The basic stiffness and strength properties of the GF/PA composite material used in this study are summarized in Table 1. The printed specimen was set to a solid infill pattern through the proprietary Eiger™ version 3.20.144 slicing software, yielding a nominal GF/PA ply thickness of 100 μm. The initial diameter of the composite filament was optically determined using the Zeiss Stand K Edu (Carl Zeiss Suzhou Co., Ltd., Suzhou, China) stereoscopy microscope, resulting in a value of 321.8 ± 7.3 μm, which falls within the 300 to 332 μm range reported in the state of the art [56,57,58,59,60]. Each filament consists of one thousand continuous fibers coated with the thermoplastic matrix, showing fiber- and matrix-dominated zones [57]. A single GF extracted from the composite filament exhibited a tensile modulus of 68 [58], 80 [60] (from pyrolysis), and 65 (from matrix digestion) [60] GPa, while the tensile strength was 2.4 (from pyrolysis) [60] and 2.4 (from matrix digestion) [60] GPa, which are slightly lower than the values commonly reported for conventional GFs in FRP composites [58].

The GF/PA composite filament has a fiber volume fraction (Vf) between 32% and 39% [57,58,60]. A single GF within the GF/PA composite filament was measured using a Jeol JSM-IT300 (JSM-IT300, JEOL Ltd., Tokyo, Japan) scanning electron microscope (SEM), yielding a fiber diameter of 9.07 ± 0.84 µm. These values fall within the reported individual fiber diameters—between 8.8 and 10 μm [56,57,58,59,60,65]. The porosity of the printed specimen has been reported to be in the range of 12.3% to 17% [18,58]. In line with the microstructural filament observations, the deposited plies exhibited fiber-dominated zones, matrix-dominated zones, and flaws.

Initially, the GF/PA specimen for the UD DCB test was designed in accordance with the ISO-15024 standard [66]. Based on the findings reported by Santos et al. [41], the CF/PA specimens used for determining the mode I and II fracture responses should have had each beam arm thickness equal to 1.5 mm, yielding a total specimen thickness of 3 mm, which would ensure proper characterization with smooth and stable crack propagation during the entire test. Consequently, a thin GF/PA DCB specimen geometry, consisting of 30 deposited GF/PA deposited layers, was imported into the Eiger™ software through the stereolithography (STL) file.

**Figure 1:**
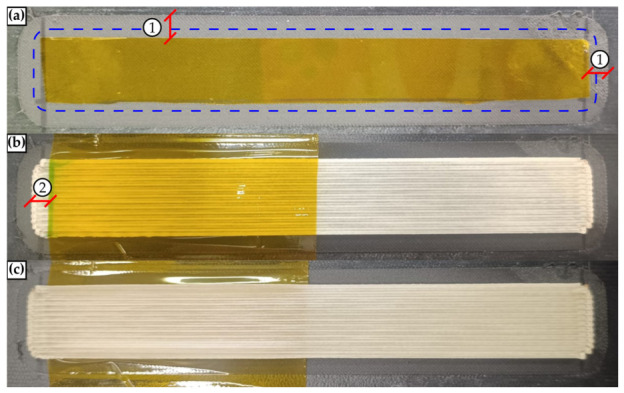
Top view of the manufacturing procedure for the GF/PA DCB specimens, showing (**a**) placement of Kapton^®^ on the 4th deposited Onyx^TM^ layer and the 9 mm distance (1) between the tape edges and the specimen borders; (**b**) additional 6 mm laminate length (2) beyond the pre-crack region to promote subsequent deposition layers; (**c**) final printed specimen.

Table 2 summarizes the printing parameters set by the Eiger™ software during the printing process of the GF/PA DCB specimens. The Eiger™ software stipulated that the floor and top plies must be printed using either a PA filament or Onyx™ (Markforged^®^, Watertown, MA, USA) composite filament. The system imposed four floor and four top Onyx™ layers. Once the 4th Onyx layer was deposited on the laminate, the printing process was paused. Rectangular Kapton^®^ tape (179 × 19 mm) with a thickness of 50 μm was placed on the printed specimen (see Figure 1a). This placement needed both careful manual alignment and visual inspection of the surface to ensure the proper positioning of the Kapton^®^ tape and the absence of wrinkles in the tape (wrinkles can generate fiber nozzle blockages during the deposition process), respectively. To support the deposition of the subsequent GF/PA layers, represented by the region delimited by the dashed blue line in Figure 1a, a clear distance of 9 mm between the specimen borders and the tape edges was defined (see Figure 1a). This manufacturing procedure allowed the removal of the floor Onyx™ layers from the GF/PA specimen without requiring a cutting process. The four top Onyx™ layers were not printed, since the printing process was stopped before their deposition. The outer wall of the printed part was defined as zero, avoiding additional specimen preparation after printing. Consequently, all Onyx^TM^ layers were removed from the GF/PA DCB specimen.

Considering the large amount of fibers aligned along the longitudinal direction of the specimen, all specimens were individually printed with rounded corners and a regular Onyx™ brim generated in the Eiger™ software. These features were implemented to reduce residual thermal stress and to avoid adhesion problems between the printing platform and the specimen due to warping effects during manufacturing. To generate the pre-crack at the mid-plane of the laminate (after the 19th GF/PA layer was deposited), the printing process was paused. Again, Kapton^®^ tape was placed on the printed specimen (see Figure 1b), which acted as a delamination initiator or artificial starter crack. As noted before, special attention was paid to avoiding wrinkles and/or misalignment of the tape. An additional 6 mm laminate length without tape beyond the pre-crack area was left to ensure the proper deposition of the subsequent fiber layers. This extra material was cut with a diamond saw, allowing controlled crack propagation during the DCB test. Following manufacturing, the printing process was resumed, and the top half of the specimen was built until the total specimen thickness was reached (see Figure 1c).

All printed specimens were stored in a Pelican^®^ dry box containing desiccant bags at room temperature until they were instrumented and tested. This procedure prevented moisture absorption by the PA matrix and avoided any possible influence on the mechanical characterization, including reductions in stiffness and strength, as well as increases in impact resistance and ductility [70,71,72]. The final dimensions of the GF/PA DCB specimens after post-processing were 175 × 25 × 3 mm (length × width × thickness), with a pre-crack length of 50 mm. A total of five DCB specimens were successfully manufactured, prepared, and tested.

### 2.2. Testing

**Figure 2:**
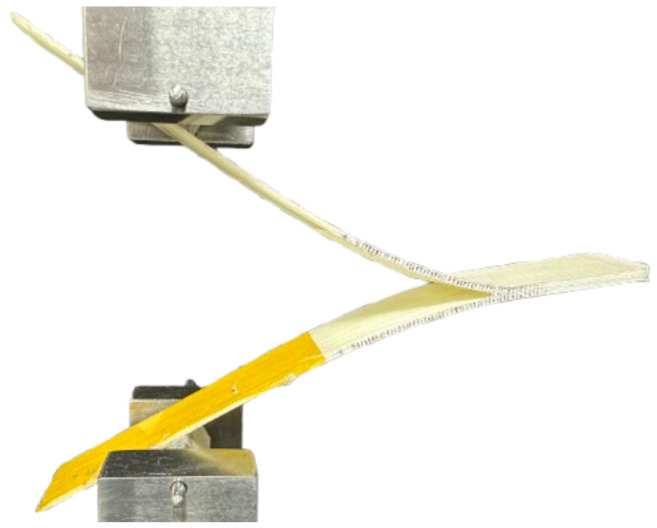
Post-processed GF/PA DCB specimen mounted in the loading fixture during mode I interlaminar fracture toughness testing.

To monitor crack propagation during testing, the edges of the specimens were slightly sanded and marked with the crack propagation lengths through vertical lines according to the guidelines of the standard. The area designated for the loading blocks on the top and bottom arms of the DCB specimens was abraded using 100-grit sandpaper to create longitudinal and transverse surface roughness, improving the adhesion of the loading blocks. Loading blocks composed of AISI/ASTM O1 tool steel were adhered to the bottom and top bending arms by applying the Henkel Loctite 401^®^ adhesive to the prepared surfaces. This adhesive was cured at room temperature for 24 h using manual clamping pressure to ensure the proper fixation of the loading blocks to the specimens. The post-processed GF/PA DCB specimen for testing is presented in Figure 2. The interlaminar fracture toughness, referred to as (*G*_I_), was determined using the modified compliance calibration (MCC) and modified beam theory (MBT) methods.

All interlaminar fracture tests were carried out using a Shimadzu AGS-300kNX (Shimadzu Corp., Kyoto, Japan) universal testing machine equipped with a 20 kN load cell at room temperature. The crosshead speed was set to 1 and 10 mm/min for loading and unloading, respectively. The minimum loading rate was selected (within the range of 1 to 5 mm/min as stated in the standard) since, during preliminary controlled crack propagation tests, we observed a more stable crack propagation response.

The initial pre-crack was extended by between 3 and 5 mm from the insert under mode I loading prior to the interlaminar fracture toughness characterization. This pre-crack extension test was conducted to ensure crack initiation from a sharp crack tip, avoiding any possible influence of the thick Kapton^®^ tape, which should not exceed 13 µm according to the ISO-15024 standard [66]. All characterization tests recorded force and displacement data. Crack propagation was monitored and recorded using a high-resolution Nikon D7500 (Nikon Corp., Tokyo, Japan) digital reflex video camera.

## 3. Results

This section presents the experimental results obtained from the mode I interlaminar fracture toughness characterization for the UD GF/PA DCB specimens.

### Interlaminar Fracture Toughness

**Figure 3:**
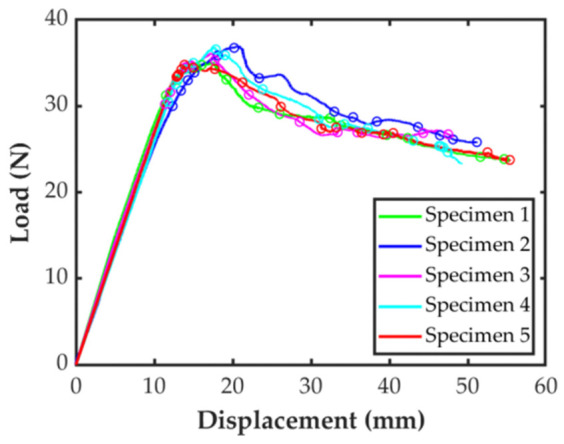
Load–displacement curves for the tested GF/PA DCB specimens. Hollow circle marks denote crack propagation lengths stated in the standard.

The load–displacement curves for all tested DCB specimens are presented in Figure 3. The hollow circle marks on the curves correspond to the measured crack propagation lengths as indicated in the ISO-15024 [66] standard. All curves experienced very similar trends and repetitiveness within the linear elastic region, showing very similar stiffness behavior until the onset of the crack. The first crack growth increments were depicted close to the peak load, after which they displayed a clear non-linear softening response along the crack propagation until the end of the test.

All experimental tests experienced smooth and stable crack propagation during the entire opening load. It is worth noting that Specimen 2 showed a secondary slope within the linear elastic region at an applied load of approximately 22 N. This specimen also exhibited the highest load-carrying capacity along the FPZ compared to the other specimens in the batch (see Figure 3). However, Specimen 3 showed a slightly higher load-carrying capacity than Specimen 2 between the applied displacements of 44 and 48 mm.

The thin DCB specimens enabled stable and controlled crack propagation during most tests. However, the final crack growth increments showed rapid crack propagation. These unstable crack propagation events were not considered in the analysis, and the load–displacement curves in Figure 3 only reflect the stable crack propagation regime. On average, Specimens 1, 2, and 3 did not achieve the final five measured crack length levels under stable crack propagation conditions, while Specimens 4 and 5 did not reach the last two. Approximately 80% of the crack propagation lengths specified in the standard were achieved under stable response conditions, allowing the reliable determination of both the *G*_Ic,ini_ and *G*_Ic,prop_ fracture toughness values of the GF/PA specimens, as shown in Table 3.

## 4. Discussion

This section analyzes the experimental results obtained for the mode I interlaminar fracture toughness for the UD GF/PA composite material. A fractographic analysis of representative post-mortem specimens was conducted to enhance the knowledge of the failure modes experienced during testing through detailed morphological observations. Moreover, the interlaminar fracture toughness values determined in this study are compared with the initiation and propagation fracture toughness reported in the literature using both composite filaments and FDM technology from Markforged^®^ (Watertown, MA, USA).

### 4.1. Fracture Toughness and Resistance Curve

**Figure 4:**
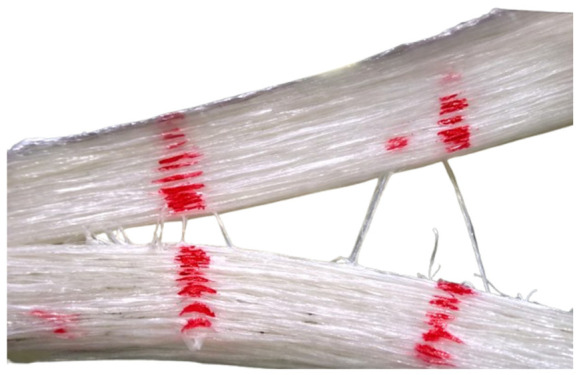
Close-ups of the crack tip areas during tests with the appearance of sporadic fiber bridging.

All specimens experienced sporadic fiber bridging events during the entire test (Figure 4). These events could increase the propagation toughness values as they increase the resistance against further delamination, raising the crack propagation resistance curve (R-curve). Fiber bridging is a common event in UD DCB tests and is considered a non-representative phenomenon. In line with these testing criteria, fiber bridging during opening is an intrinsic mechanical characteristic of this commercial printable composite material from Markforged^®^ [34,41,44,47,73].

**Figure 5:**
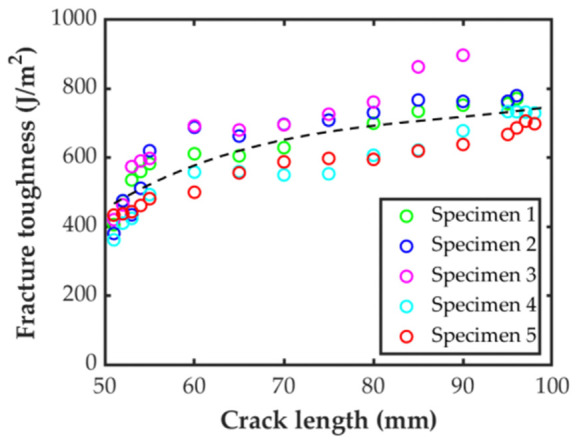
Crack propagation resistance curve for the GF/PA DCB specimens. Black dashed line corresponds to a polynomial trendline fitted to the tested specimen batch.

The R-curve for the GF/PA DCB specimens can be seen in Figure 5. All specimens experienced a similar scatter in fracture toughness along the crack length. In good agreement with the observations described in Section 3, the two largest crack propagation lengths of Specimen 3 exhibited higher fracture toughness values than the other specimens in the batch. In general, the fracture toughness experienced a rising trend with increasing crack lengths. A pronounced initial increase in resistance was observed between crack lengths of approximately 51 and 65 mm, followed by a second region showing more gradual growth in resistance up to the end of crack propagation. The *G*_Ic,ini_ values of the GF/PA material determined using the MMC and MBT methods exhibited very similar trends (Table 3), with a difference of less than 4% between them. In line with this toughness value determination, *G*_Ic,prop_ exhibited virtually identical values between both methods.

### 4.2. Fractographic Analysis

A fractographic analysis of the representative GF/PA DCB post-mortem specimens was carried out after testing. All specimens were manually split open, and the first 10 mm of the crack propagation length was removed using the same diamond saw (Section 2), avoiding morphological contamination generated during the cutting operation. The same (see Section 2.1) Jeol JSM-IT300 SEM was used to analyze the microscopic failure characteristics under an accelerating voltage of 20 kV. To enhance the scanning quality prior to the observations, a thin gold coating was deposited on the underside surface of each specimen using the Cressington 108 auto sputter coater. Subsequently, each specimen was mounted on the SEM specimen holder using a carbon tape.

**Figure 6:**
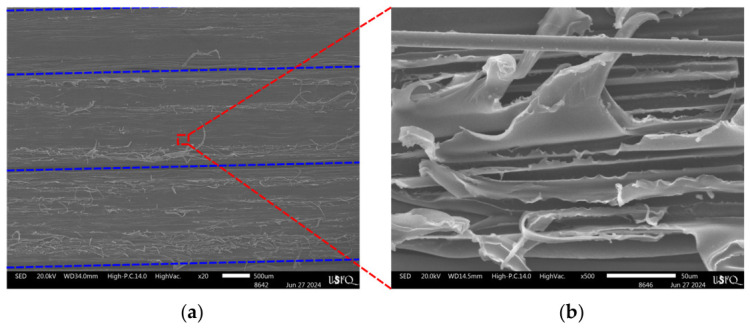
SEM image analysis of the fracture surface of a representative GF/PA DCB specimen. Dashed blue lines indicate the adjacent deposited filament raster paths. (**a**) Low magnification; (**b**) high magnification showing traces of pull-out fibers.

The fracture surface at low magnification (see Figure 6a) showed the smoothest morphology, with a darker region located in the upper part of the image. The surface roughness increased at the middle and lower parts of the image, showing permanent matrix deformation in brighter areas. The adjacent deposited filament rasters formed shallow longitudinal furrows with darker areas, which are depicted with dashed blue lines in Figure 6a. These fiber beads exhibited dry fiber zones, polymeric matrix-dominated zones, and flaws. Furthermore, the deposited glass fibers did not exhibit perfectly straight alignment; instead, they exhibited a noticeable waviness pattern along the fracture propagation path.

To further improve the understanding of the dominant failure mechanisms, the fracture surface was observed at higher magnification (see Figure 6b). The brighter regions associated with the permanent plastic deformation of the polymeric matrix exhibited ridge shapes. A few traces of pulled-out fibers were observed. Again, fiber- and matrix-dominated zones were identified across the micrograph. Moreover, a long debonded fiber extending across the width of the micrograph was observed at a higher morphological level. This fiber was not aligned with the surrounding traces of pulled-out fibers at a lower level.

**Figure 7:**
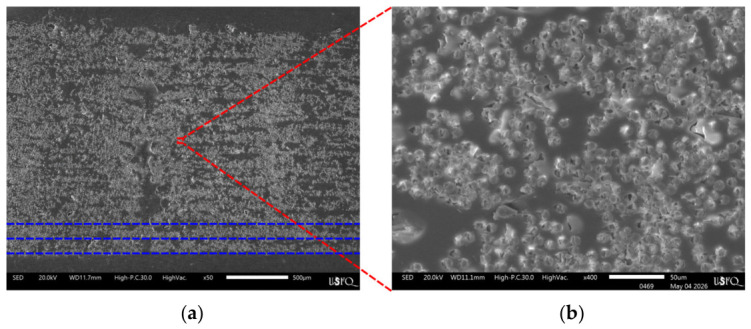
Scanning electron microscopy micrographs of the cross-sections of a representative tested DCB specimen. (**a**) Low magnification; (**b**) high magnification, showing the random distribution of void, matrix, and fiber zones.

The cross-sectional morphological analysis of one beam of a representative post-mortem tested DCB specimen was carried out by following the same SEM preparation procedure described in Section 4.2. Figure 7a clearly shows a random distribution of fiber-dominated regions in brighter areas, matrix-dominated regions in darker zones, and voids enclosed within irregular geometries. The formation of such voids may be attributed to insufficient filling pressure from the fiber nozzle in the printing head, limited pushing force from the fiber feeding mechanism during the deposition process, and the absence of a post-processing compaction stage [34,44,45,51]. The bonding between consecutive deposited layers—the first two plies highlighted by dashed blue lines in Figure 7a—indicates good interfacial adhesion quality for this type of additive composite material, revealing no evident interface traces along the entire laminate.

The microstructural characterization performed on the cross-sectional micrographs revealed a GF Vf range of 30.53 ± 3.19%, PA volume fraction content of 67.03 ± 0.97%, and a void density of 2.40 ± 1.44%. Characterization indicated attractive limited content of flaws; interestingly, this value was lower than those reported in the literature of 12.3% and 17% [18,58] (as previously noted in Section 2.1), suggesting low repetitiveness for this printed composite material. In contrast, the fiber content was in good agreement with the values reported by Chabaud et al. [58].

The cross-sectional micrographs at higher magnification in Figure 7b depict the random distribution of individual fibers appearing in brighter regions, embedded within the PA matrix in darker areas, and the limited presence of voids with irregular geometries. These morphological features reflect characteristics similar to those observed at lower magnification. The fibers predominantly showed a circular geometry with slight morphological distortions. In addition, this micrograph exposed good PA impregnation around all fibers.

### 4.3. Fracture Toughness Comparison

Finally, to place the findings of the present investigation into a broader perspective, the mode I interlaminar fracture toughness values obtained in this study were compared with those reported in the literature for UD laminates using commercial continuous-fiber composite filaments from Markforged^®^. Most studies focused on the CF/PA-reinforced system, while a limited number of studies have analyzed KF/PA and HSHTGF/PA, as noted before (Section 1).

**Figure 8:**
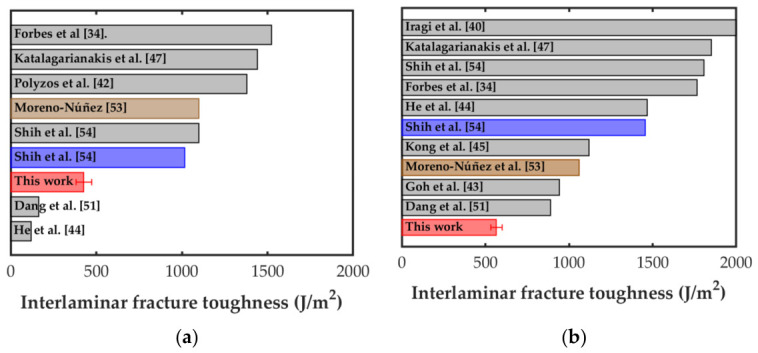
Quantitative comparison of the mode I interlaminar fracture toughness of the GF/PA composite material with values reported in the literature for CF/PA in grey bars, KF/PA in brown bars, and HSHTGF/PA in blue bars. The work by Goh et al. [43] did not use a Markforged^®^ 3D printer. (**a**) shows crack initiation, while (**b**) shows propagation.

Figure 8a summarizes the reported G_Icini_ values for CF/PA, KF/PA, and HSHTGF/PA by means of light gray, light brown, and light blue bars, respectively. A pronounced scatter was observed between the reported CF/PA toughness values, denoting significant variability and reduced repeatability for this additive composite material [14,48,74,75,76]. Interestingly, Forbes et al. [34] (1524 J/m^2^), Katalagarianakis et al. [47] (1440 J/m^2^), and Polyzos et al. [42] (1380 J/m^2^) obtained similar onset fracture toughness values, corresponding to the highest ones. On the other hand, He et al. [44] reported the lowest onset fracture toughness value (118.5 J/m^2^) for non-post-processed tested specimens, followed by the value of 163 J/m^2^ reported by Dang et al. [51]. The G_Icini_ value obtained for the DCB GF/PA specimens in this study lies between the high [34,42,47,54] and low [44,51] fracture toughness bounds of this comparative database. Moreover, the G_Icini_ values for KF/PA and HSHTGF/PA reported by Moreno-Núñez et al. [53] (visually assessed as 1100 J/m^2^) and Shih et al. [54] (1015 J/m^2^) were higher than the GF/PA one (Table 3).

Similarly to the observations of the reported values of onset fracture toughness, the G_Ic,prop_ values for CF/PA systems also exhibited a scatter pattern between investigations (see Figure 8b). Three distinct response groups were identified. The first group, corresponding to the lower bound, includes the studies by Dang et al. [51] (889 J/m^2^) and Goh et al. [43] (943 J/m^2^). Kong et al. [45] and He et al. [44] reported intermediate G_Ic,prop_ values for CF/PA of 1120 and 1467 J/m^2^, respectively; these two values can be considered as a transition between the lower and upper bounds of the database. The third group exhibited the highest toughness values, including those reported by Forbes et al. [34] (1766 J/m^2^), Shih et al. [54] (1806 J/m^2^), Katalagarianakis et al. [47] (1850 J/m^2^), and Iragi et al. [40] (2000 J/m^2^). In this context, the G_Ic,prop_ values of the GF/PA DCB specimens in this study were lower than those of all CF/PA systems within these three response groups. In line with the findings of the onset fracture toughness, the propagation toughness values reported for HSHTGF/PA by Shih et al. [54] (1457 J/m^2^) and for KF/PA by Moreno-Núñez et al. [53] (1060 J/m^2^) were higher than the GF/PA one in the present study (Table 3).

The limited propagation fracture toughness value of the DCB GF/PA specimens might be attributed to their low flexural moduli and flexural strength, ranging between 14.7 and 22 GPa and 149 and 205 MPa [61,62,64] (Table 1), respectively. The CF/PA composite material exhibited a flexural modulus ranging between 35.8 and 45.8 GPa and flexural strength ranging from 430 to 485 MPa [13,14,44,61,64]. On average, the flexural performance of CF/PA was more than two times that of GF/PA in terms of both the flexural strength and flexural stiffness.

Interestingly, the GF/PA system exhibited average flexural stiffness and flexural strength values that were approximately 56% and 11% higher, respectively, than those reported for the KF/PA composite material in the literature (6.65 [56] and 14.1 GPa [61]; 125.8 [56] and 189.8 MPa [61]). However, according to the composite material supplier [62], the KF/PA system exhibited superior out-of-plane mechanical properties to the GF/PA one, showing flexural strength of 240 MPa and a flexural modulus of 26 GPa. This superior flexural strength is even more pronounced for HSHTGF/PA composites, with 420 MPa.

Overall, this study demonstrates the successful manufacturing, preparation, and experimental characterization of an additively manufactured GF/PA composite material under mode I interlaminar fracture toughness testing, following established guidelines for traditional laminated composite materials and experimental findings reported in the literature. To the best of the authors’ knowledge, this is the first work to characterize GF/PA DCB specimens without the use of doublers or thick specimen configurations, avoiding the potential overestimation of the fracture resistance capacity. It is worth noting that the previous comparative analysis of fracture toughness values reported in the state of the art (Figure 8) provides a broader outlook on the performance of different continuous fiber-reinforced thermoplastic systems using Markforged^®^ composite filaments and its FDM technology.

## 5. Conclusions

This study provides the first systematic experimental campaign to characterize the initiation and propagation interlaminar fracture toughness of the UD GF/PA composite material using the on-desktop MarkTwo^®^ 3D printer from Markforged^®^, addressing the existing gap in fracture toughness data for GF-reinforced systems. Individual thin GF/PA DCB specimens were manufactured according to previous experimental findings reported in the literature. The artificial starter crack was created using Kapton^®^ adhesive tape during the printing process, reducing the need for additional cutting operations during the subsequent DCB specimen preparation stage. Sporadic fiber bridging events, together with a stable and smooth crack propagation mode, were observed during the controlled crack propagation test, validating the reported results of the present study. The R-curve exhibited a progressive increase in fracture resistance along the crack propagation length, characterized by distinct slopes. The onset interlaminar fracture toughness mode I values were in good agreement between the MCC and MBT methods, while the reported propagation fracture toughness values were virtually identical for both methods. The observed fracture surface of a representative post-mortem DCB specimen revealed certain zones with smooth and rough morphological surfaces, shallow longitudinal furrows between adjacent deposited filament rasters, matrix-dominated zones, dry fibers or poor bonding between the fibers and matrix, random flaws, and longitudinally deposited fibers exhibiting a wavy shape. The cross-sectional morphological analysis of the same representative DCB specimen revealed that the matrix contained the largest volumetric content, followed by the fibers, and voids accounted for the lowest density. All fibers showed good PA coating under higher magnification. The quantitative comparison of the fracture toughness of the GF/PA DCB specimens with the reported values for commercial Markforged^®^ composite filaments revealed that the onset fracture toughness response of GF/PA was situated between those of reported CF/PA systems and remained below those corresponding to KF/PA and HSHTGF/PA ones. The propagation fracture toughness of the GF/PA composite material exhibited lower crack growth resistance than all previously reported CF/PA, KF/PA, and HSHTGF/PA laminates. This might be attributed to the superior flexural mechanical performance of CF/PA, KF/PA, and HSHTGF/PA composites compared to GF/PA.

Future research is suggested to evaluate the effects of environmental exposure on the interlaminar fracture toughness behavior of UD laminate configurations and, even more challengingly, multidirectional laminates. The latter can incorporate mechanical characterization under mode II and MMB loading conditions to further understand the fracture responses of the final printed components.

## Figures and Tables

**Table 1:** Elastic mechanical properties of PA and GF/PA materials. *E*_11_, *E*_22_, *G*_12_, and *E_f_* denote the longitudinal, transverse, in-plane shear, and flexural modulus, respectively, whereas *X*_t_, *Y*_t_, *S*, and σ_f_ denote their corresponding strengths. The Poisson ratio is υ_12_.

*E*_11_ (GPa)	*E*_22_ (GPa)	υ_12_	*G*_12_ (GPa)	*E*_f_ (GPa)	*X*_t_ (MPa)	*Y*_t_ (MPa)	*S* (MPa)	σ_f_ (MPa)	Ref.
PA									
0.94	---	---	---	0.84	0.05	---	---	32	[61]
1.7	---	---	---	1.4	0.04	---	---	50	[62]
GF/PA									
25.9	1.1	0.37	0.9	---	574.6	9.8	67.7	---	[18]
28	0.3	---	---	---	260	10	---	---	[58]
21	5	---	1.4	---	---	---	11	---	[63]
19.6	---	---	---	16.2	381.2	---	---	205.1	[61]
7.2	---	---	---	14.7	450	---	---	149	[64]
21	---	---	---	22	590	---	---	200	[62]

**Table 2:** Printing parameters defined by Eiger™ software for manufacturing UD GF/PA DCB specimens using a MarkTwo^®^ 3D printer.

Printing Parameter	Value	Ref.
Extruder temperature (°C)	250	[57,67,68]
Fiber nozzle diameter (mm)	0.9	[67]
Printing speed (mm/s)	10, 14	[67,69]
Printing bed (°C)	Room temperature	---

**Table 3:** Initiation and propagation interlaminar fracture toughness in mode I for the GF/PA composite material.

	Interlaminar Fracture Toughness (J/m^2^)	
Data Reduction	Onset	Propagation
MCC	411 ± 46	565 ± 35
MBT	427 ± 55	566 ± 35

## Data Availability

The data presented in this study are available on reasonable request from the corresponding author. The data are not publicly available due to their large size and because they form part of ongoing research activities.
